# Deep learning for enhanced prediction of diabetic retinopathy: a comparative study on the diabetes complications data set

**DOI:** 10.3389/fmed.2025.1591832

**Published:** 2025-06-16

**Authors:** Weijun Gong, You Pu, Tiao Ning, Yan Zhu, Gui Mu, Jing Li

**Affiliations:** ^1^School of Mathematics Kunming University, Kunming University, Kunming, Yunnan, China; ^2^Department of Rehabilitation, Baoshan People’s Hospital, Baoshan, Yunnan, China; ^3^Engineering Research Center for Urban Modern Agriculture of Higher Education in Yunnan Province, School of Agriculture and Life Sciences, Kunming University, Kunming, Yunnan, China

**Keywords:** diabetic retinopathy, deep learning model, prediction models, model comparison, machine learning

## Abstract

**Background:**

Diabetic retinopathy (DR) screening faces critical challenges in early detection due to its asymptomatic onset and the limitations of conventional prediction models. While existing studies predominantly focus on image-based AI diagnosis, there is a pressing need for accurate risk prediction using structured clinical data. The purpose of this study was to develop, compare, and validate models for predicting retinopathy in diabetic patients via five traditional statistical models and deep learning models.

**Methods:**

On the basis of 3,000 data points from the Diabetes Complications Data Set of the National Center for Population Health Sciences Data, the differences in the characteristics of patients with diabetes mellitus and diabetes combined with retinopathy were statistically analyzed using SPSS software. Five traditional machine learning models and a model based on deep neural networks (DNNs) were used to train models to assess retinopathy in diabetic patients.

**Results:**

Deep learning-based prediction models outperformed traditional machine learning models, namely logistic regression, decision tree, naive Bayes, random forest, and support vector machine, on all the datasets and performed better in predicting retinopathy in diabetic patients (accuracy, 0.778 vs. 0.753, 0.630, 0.718, 0.758, 0.776, respectively; F1 score, 0.776 vs. 0.751, 0.602, 0.724, 0.755, 0.776, respectively; AUC, 0.833 vs. 0.822, 0.631, 0.769, 0.829, 0.831, respectively). To enhance the interpretability of the deep learning model, SHAP analysis was employed to assess feature importance and provide insights into the key drivers of retinopathy prediction.

**Conclusion:**

Deep learning models can accurately predict retinopathy in diabetic patients. The findings of this study can be used for prevention and monitoring by allocating resources to high-risk patients.

## 1 Introduction

Diabetes mellitus (DM), a complex metabolic disorder characterized by chronic hyperglycemia, is marked by persistently elevated blood glucose levels and impaired carbohydrate metabolism (1, 2). This condition is associated with microvascular and macrovascular complications, including damage to the kidneys, nerves, and eyes and an increased risk of cardiovascular diseases (3). The pathogenesis of diabetes mellitus is complex, with varied manifestations and progressive development (1, 4). Diabetes is a rapidly growing global health emergency in the 21st century, with approximately 536.6 million adults living with diabetes (both diagnosed and undiagnosed, type 1 and type 2) according to the International Diabetes Federation’s 2021 report (5, 6). Diabetic retinopathy (DR) is a common microvascular complication of diabetes mellitus and a leading cause of vision loss in elderly individuals (7). In the early stages of diabetic retinopathy, hyperglycemia and altered metabolic pathways lead to oxidative stress and neurodegeneration (8). Chronic hyperglycemia damages retinal capillaries, which disrupts light perception and signal transmission, ultimately leading to DR. DR is particularly common among the working-age population and is widespread globally, with an estimated 191 million cases projected by 2030 (9). Although advanced DR can result in blindness, early detection is difficult because its visual symptoms are not easily detectable. However, consistent screening and early diagnosis can potentially lower the risk of vision impairment and treatment costs by 57.0% (9). Patients diagnosed with referable DR need a thorough ophthalmologic evaluation and appropriate medical or surgical intervention to prevent vision loss.

Artificial intelligence (AI) technologies have been employed for over two decades to address the significant screening demand for diabetic retinopathy. Initially, AI methods for DR detection focused on identifying pathological signs in fundus images, such as hemorrhages, new blood vessel formation, and exudates, which were then used to assess the presence of DR (10–12). As computational capacities have improved, deep learning (DL) has emerged as the dominant AI approach in DR screening, with many deep learning models now surpassing traditional feature-based machine learning techniques (13). This technological evolution is reflected across medical imaging domains, where novel architectures continue to push diagnostic boundaries. Notably, Haq et al. developed DCNNBT, achieving 99.18% brain tumor classification accuracy through optimized convolutional layers and hyperparameter tuning (14), while Kumar et al. demonstrated 96.2% detection accuracy using transfer learning with augmented MRI data (15). Parallel advances in segmentation are exemplified by Yousef et al.’s systematic optimization of U-Net variants for brain tumor localization (16). Particularly noteworthy is the Alhussen et al. introduced XAI-RACapsNet system, which combines capsule networks with explainable AI for mammography diagnosis, addressing critical challenges in model interpretability (17). While these image-based breakthroughs showcase DL’s capabilities in radiological interpretation, our study addresses a distinct clinical need: leveraging structured electronic health records for DR prediction.

DL is a subset of machine learning that performs automatic feature learning via a multilayer algorithmic structure, an artificial neural network inspired by human neural networks (18). Recent advancements in DL have shown considerable promise in enhancing diagnostic accuracy, indicating that deep neural network (DNN) is a valuable tool for improving the early diagnosis and classification of diseases (19). For example, Moya-Albor et al. developed a DL-based method for DR classification, employing knowledge distillation (KD) strategies to improve model performance on imbalanced datasets (20). Similarly, Lombardo et al. explored sex differences in cardiovascular complications among diabetic patients using three-dimensional contingency table analysis (21). Rehman et al. emphasized the importance of handling missing blood glucose data for predicting postprandial hypoglycemia, finding that random forest models were robust to missing data (22). Tašić et al. proposed a fuzzy multi-layered system for predicting type 2 diabetes risk by incorporating physical, behavioral, and environmental factors, aiding doctors in assessing patient risk more accurately (23). Furthermore, Moya-Albor et al. introduced a bio-inspired watermarking method for privacy protection in medical image analysis without compromising diagnostic quality (24). Additionally, Neamtu et al. revealed the crucial role of disease features, complications, and socioeconomic factors in blood glucose control prediction for children with type 1 diabetes, using machine learning algorithms (25).

Several developed countries have established DR screening programs aimed at the early diagnosis, monitoring, and timely treatment of DR (26). However, the diagnostic accuracy may not be optimal, and there is a paucity of relevant research.

Therefore, the purpose of this study was to assess the discriminative accuracy of a deep learning-based prediction model for diabetes and diabetic-complicated retinopathy using the Diabetes Complications Data Set of the National Center for Population Health Sciences Data (27) and compare it with five traditional machine learning models. The key novelty of this study lies in its integration of deep learning techniques with a robust feature learning framework to predict DR more accurately than traditional statistical and machine learning models. By systematically comparing the performance of deep neural networks with five classical models, this study demonstrates the superior predictive power of DL in handling complex, high-dimensional data from diabetic patients. Furthermore, the findings highlight the potential of DL to inform targeted interventions and resource allocation for high-risk populations, ad-dressing gaps in existing DR screening programs and contributing to the early detection and management of DR.

## 2 Materials and methods

### 2.1 Study design and population

This research employed the Population Health Scientific Data Warehouse (PHDA), which focuses on managing scientific data in the area of population health derived from scientific and technological projects supported by national governmental funding. It also includes data processed by partnering institutions to meet specific requirements and data produced by various institutions and individuals.

**FIGURE 1:**
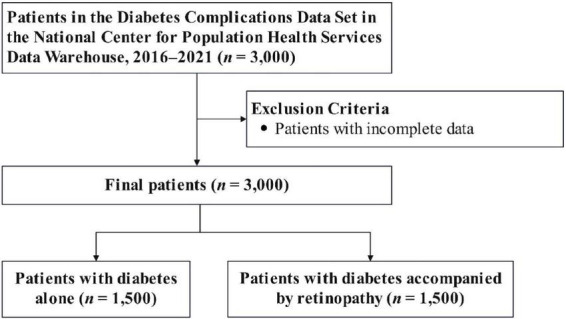
Flowchart showing the selection of the study population.

The detailed study design is illustrated in Figure 1. We utilized the Diabetes Complications Data Set (DCDS), which contained 3,000 data points. All data points had complete information; thus, no exclusions were necessary. The dataset included 1,500 individuals with diabetes and 1,500 with diabetes complicated by retinopathy. Statistical analyses were performed on these groups.

This study received approval from the National Population Health Sciences Data Center-Clinical Medical Center, and obtain a license to use the data^1^. Since this was a retrospective analysis, informed consent was not needed. Owing to legal and ethical considerations, the authors are unable to share the dataset publicly. The data can be accessed through a formal request to the National Center for Population Health Sciences Data Warehouse.^2^

### 2.2 Research variables

In this study, we utilized data from the PHDA to explore the factors associated with diabetes and diabetes-related retinopathy. The analysis incorporated variables namely, the latest age, sex, and diabetes classification. Additionally, body mass index (BMI), fasting blood glucose (GLU), systolic blood pressure (SBP), diastolic blood pressure (DBP), total cholesterol (TC), blood urea (BU), hemoglobin (HB), total bilirubin (TBILI), globulin (GLO), and other physical activity measures were examined. To account for medical history, we analyzed the frequency of hospital visits, prescribed medications, and duration of hospital stays over the past 2 years.

### 2.3 Algorithm development and statistical analysis

The data preprocessing pipeline comprised three sequential steps: (1) exclusion of variables with over 50% missing values to ensure data quality; (2) univariate feature selection using chi-square tests for categorical variables and independent *t*-tests for continuous variables (significance threshold *p* < 0.05); and (3) normalization of all continuous variables to zero mean and unit variance using Z-score transformation, along with binary (0/1) or one-hot encoding of categorical variables.

**FIGURE 2:**
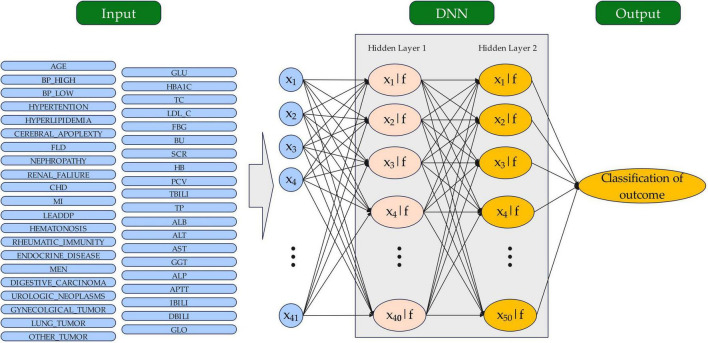
The structure of the DNN model for outcome classification. The circles represent neurons. DNN, sdeep neural network.

Deep neural networks (DNNs) are a type of artificial neural network (ANN) with multiple hidden layers between input and output layers, enabling them to learn complex features from data through hierarchical abstraction. Inspired by the human brain, DNNs process data via interconnected neurons, each applying weighted inputs and non-linear activation functions. DNNs excel at analyzing large, high-dimensional datasets; thus, they are ideal for tasks such as image recognition, natural language processing, and healthcare predictions, including for diabetic retinopathy. In this study, a predictive model for diabetic retinopathy was developed using a DNN approach. The data processing pipeline for the DNN model is illustrated in Figure 2. The dataset was randomly divided into a training set and a validation set at a 7:3 ratio to enhance the model’s learning and prediction accuracy for unseen data (28, 29). The DNN architecture consisted of an input layer (41 features), two hidden layers (40 and 50 nodes with rectified linear unit (ReLU) activation), and a sigmoid output layer, ensuring progressive abstraction of high-level features from the data. We applied a ReLU activation function in the hidden layer. ReLU is a widely used function that introduces nonlinearity by outputting the input directly if it is positive and zero otherwise, allowing the model to learn complex patterns more effectively. This choice enhances the model’s capacity to handle intricate relationships in the data.

To mitigate the risk of overfitting, we implemented a dropout rate of 0.2 during training, randomly disregarding 20% of the neurons in each iteration. This approach aids in improving the model’s generalization capabilities. The model was optimized using the Adam optimizer, a widely used algorithm that combines momentum and adaptive learning rates. Adam dynamically adjusts each parameter’s learning rate based on gradient moment estimates. This approach enhances efficiency and makes the optimizer particularly suitable for large, noisy datasets. In this study, the Adam optimizer was employed with a learning rate of 0.00001, which was determined through systematic evaluation of multiple candidate values (1e-4, 1e-5, and 1e-6) on the validation set. Alternative optimizers, such as stochastic gradient descent (SGD), RMSprop, or Adagrad, could also be considered. However, Adam is particularly suitable for this setting due to its ability to handle sparse gradients and adaptively adjust learning rates, making it robust and effective for complex models like DNNs.

Additionally, we incorporated an early stopping mechanism that monitors validation accuracy and halts training if no significant improvement occurs within 80 consecutive epochs, thus conserving computational resources and preventing overfitting. For model evaluation, we utilized various performance metrics, including accuracy, precision (positive predictive value), recall (sensitivity), and the area under the ROC curve (AUC), which collectively reflect the classification performance. The optimal classification threshold was determined via the Yuden index, which accounts for sensitivity and specificity to establish the best decision boundary. All the statistical analyses were performed via SPSS (version 26) and Python (version 3.8.0), with two-tailed *p* values; a *p* value of less than 0.05 was considered to indicate significance.

The deep learning model was interpreted utilizing Python’s SHAP module (version 0.38.1). The SHAP plots functioned as a crucial resource for understanding machine learning models (30). In these plots, the width of the horizontal axis linked to each variable denoted its impact on the outcome, while the dot colors illustrated the strength of that influence. This methodological framework establishes a solid foundation for developing and evaluating deep learning models, ensuring the reliability and validity of the findings.

### 2.4 Computational implementation

The model was trained on a laptop (Intel Core i7-13700H, 16GB RAM; NVIDIA GeForce RTX 4060 GPU) using TensorFlow with CUDA 12.7 acceleration. Dynamic GPU memory allocation, batch processing (64 samples/batch), and early stopping (patience = 80 epochs) enabled efficient training, completing 200 epochs in approximately 6.6 h with modest resource utilization (peak VRAM: 1.1GB/8GB)

## 3 Results

### 3.1 Patient characteristics

The study analyzed 3,000 individuals diagnosed with diabetes, consisting of 1,500 with diabetes alone and 1,500 with diabetes accompanied by retinopathy. The DCDS dataset included 88 variables; independent samples were assessed via chi-square tests and t tests. Significant differences were identified across most variables, except for SEX, NATION, MARITAL_STATUS, BUN, CP, and INS. The baseline characteristics of all participants are presented in Table 1.

**TABLE 1:** Baseline characteristics of the study participants in diabetes alone and diabetes with retinopathy groups.

Variables	Presence of diabetes with retinopathy			Variables	Presence of diabetes with retinopathy		
	No (*n* = 1,500)	Yes (*n* = 1,500)	*p*-value		No (*n* = 1,500)	Yes (*n* = 1,500)	*p*-value
AGE	58.99 ± 11.24	56.59 ± 10.94	< 0.001	IBILI	7.84 ± 5.13	7.03 ± 3.88	< 0.001
BP_HIGH	135.00 ± 19.92	142.36 ± 21.42	< 0.001	GLO	26.45 ± 4.95	25.74 ± 4.81	< 0.001
BP_LOW	78.96 ± 11.88	81.97 ± 11.81	< 0.001	CHD			< 0.001
GLU	8.20 ± 3.69	8.69 ± 4.08	0.001	No	1126 (75.10)	889 (59.30)	
HBA1C	7.44 ± 1.52	8.15 ± 1.87	< 0.001	Yes	374 (24.90)	611 (40.70)	
TC	4.48 ± 1.31	4.75 ± 1.51	< 0.001	MI			< 0.001
LDL_C	2.73 ± 1.02	2.97 ± 1.24	< 0.001	No	1433 (95.50)	1377 (91.80)	
FBG	6.45 ± 35.48	9.32 ± 41.56	0.043	Yes	67 (4.50)	123 (8.20)	
BU	6.04 ± 3.68	8.19 ± 5.78	< 0.001	LEADDP			< 0.001
SCR	85.24 ± 89.99	127.93 ± 139.18	< 0.001	No	1143 (76.20)	1381 (92.10)	
HB	136.30 ± 21.44	127.11 ± 23.83	< 0.001	Yes	357 (23.80)	119 (7.90)	
PCV	0.40 ± 0.06	0.37 ± 0.07	< 0.001	HEMATONOSIS			< 0.001
TBILI	12.19 ± 15.52	9.77 ± 5.35	< 0.001	No	1173 (78.20)	1383 (92.20)	
DBILI	4.35 ± 12.40	2.73 ± 1.86	< 0.001	Yes	327 (21.80)	117 (7.80)	
TP	67.31 ± 6.57	63.66 ± 7.84	< 0.001	RHEUMATIC_ IMMUNITY			0.004
ALB	40.85 ± 5.15	37.92 ± 6.20	< 0.001	No	1463 (97.50)	1434 (95.60)	
ALT	27.56 ± 35.44	20.54 ± 15.50	< 0.001	Yes	37 (2.50)	66 (4.40)	
AST	22.87 ± 30.01	17.81 ± 9.55	< 0.001	ENDOCRINE_ DISEASE			< 0.001
GGT	50.39 ± 86.79	34.81 ± 47.90	< 0.001	No	896 (59.70)	1102 (73.50)	
ALP	77.65 ± 55.85	72.72 ± 31.56	0.003	Yes	604 (40.30)	398 (26.50)	
APTT	36.96 ± 8.56	36.42 ± 5.19	0.036	MEN			0.031
HYPERTENSION			< 0.001	No	1457 (97.10)	1435 (95.70)	
No	407 (27.10)	547 (36.50)		Yes	43 (2.90)	65 (4.30)	
Yes	1093 (72.90)	953 (63.50)		DIGESTIVE_ CARCINOMA			< 0.001
HYPERLIPIDEMIA			< 0.001	No	1466 (97.70)	1381 (92.10)	
No	1251 (83.40)	1093 (72.90)		Yes	34 (2.30)	119 (7.90)	
Yes	249 (16.60)	407 (27.10)		UROLOGIC_ NEOPLASMS			0.019
CEREBRAL_ APOPLEXTY			< 0.001	No	1491 (99.40)	1478 (98.50)	
No	1352 (90.10)	1424 (94.90)		Yes	9 (0.60)	22 (1.50)	
Yes	148 (9.90)	76 (5.10)		GYNECOLGICAL_ TUMOR			< 0.001
FLD			0.013	No	1470 (98.00)	1431 (95.40)	
No	1000 (66.70)	1063 (70.90)		Yes	30 (2.00)	69 (4.60)	
Yes	500 (33.30)	437 (29.10)		LUNG_TUMOR			< 0.001
NEPHROPATHY			< 0.001	No	1491 (99.40)	1454 (96.90)	
No	597 (39.80)	1126 (75.10)		Yes	9 (0.60)	46 (3.10)	
Yes	903 (60.20)	374 (24.90)		OTHER_TUMOR			< 0.001
RENAL_FALIURE			< 0.001	No	1427 (95.10)	1327 (88.50)	
No	1341 (89.40)	1476 (98.40)		Yes	73 (4.90)	173 (11.50)	
Yes	159 (10.60)	24 (1.60)					

BP, blood pressure; GLU, glucose; HBA1C, hemoglobin A1c; TC, total cholesterol; LDL_C, low-density lipoprotein cholesterol; FBG, fasting blood glucose; BU, blood urea; SCR, serum creatinine; HB, hemoglobin levels; PCV, packed cell volume; TBILI, total bilirubin; DBILI, direct bilirubin; TP, total protein; ALB, albumin; ALT, alanine aminotransferase; AST, aspartate aminotransferase; GGT, gamma–glutamyl transferase; ALP, alkaline phosphatase; APTT, activated partial thrombin time; IBILI, indirect bilirubin; FLD, fatty liver disease; IBILI, indirect bilirubin; GLO, globulin; CHD, coronary heart disease; MI, myocardial infarction; LEADDP, lower extremity disease of diabetes patients; MEN, multiple endocrine neoplasia.

Patients with diabetes and retinopathy were younger (56.59 ± 10.94 vs. 58.99 ± 11.24 years), had higher hemoglobin A1c (HBA1C) levels (8.15 ± 1.87 vs. 7.44 ± 1.52), lower hemoglobin levels (HB) (127.11 ± 23.83 vs. 136.30 ± 21.44), and a higher proportion of nephropathy (60.20% vs. 24.90%). More of these patients had lower extremity atherosclerotic disease with diabetic peripheral polyneuropathy (LEADDP) (23.80% vs. 7.90%) and hematonosis (21.80% vs. 7.80%). Conversely, these patients had lower incidences of myocardial infarction (MI) (4.50% vs. 8.20%), hyperlipidemia (16.60% vs. 27.10%), and coronary heart disease (CHD) (24.90% vs. 40.70%). In addition, the levels of albumin (ALB), alanine aminotransferase (ALT), aspartate aminotransferase (AST), gamma-glutamyl transferase (GGT), alkaline phosphatase (ALP) and activated partial thromboplastin time (APTT) were lower in the retinopathy group than in the diabetes alone group.

### 3.2 Results of the DNN prediction model

**FIGURE 3:**
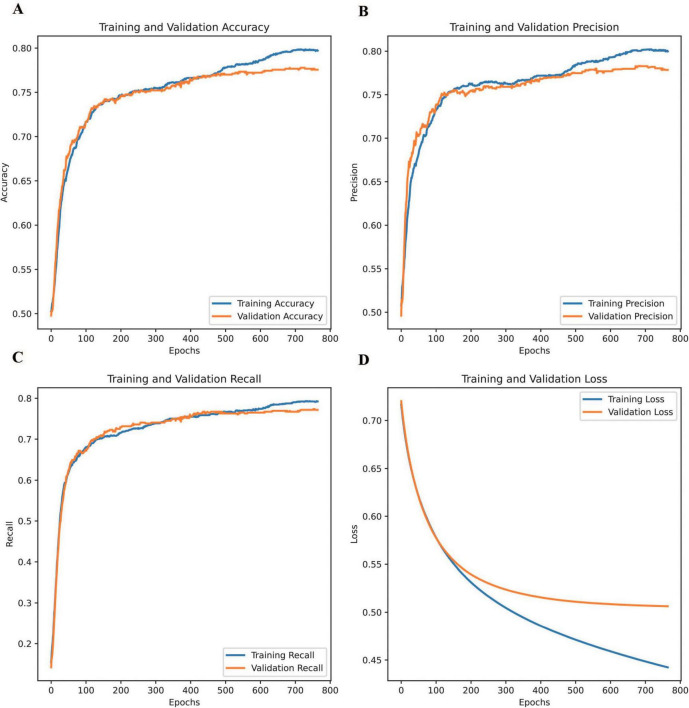
Changes in the accuracy, precision, recall, and loss of the DNN during training and validation. **(A)** training and validation accuracy, **(B)** training and validation precision, **(C)** training and validation recall, and **(D)** training and validation loss.

Figure 3 shows the changes in DNN accuracy, precision, recall, and loss during training and validation. The DNN demonstrated significant improvements across key performance metrics throughout the training process, evaluated in accordance with the standardized indices for ophthalmic AI models (e.g., sensitivity, specificity, AUC) recommended by the Guidelines on clinical research evaluation of artificial intelligence in ophthalmology (2023) (31).

Training accuracy, precision, and recall were calculated using the formulae defined in Section “Commonly-used indices and formulae for ophthalmic artificial intelligence diagnostic model evaluation” of the Guidelines (31), highlighting the model’s ability to learn effectively from the training dataset and capture underlying patterns. Validation accuracy followed a similar trend, plateauing slightly earlier than training accuracy and remaining marginally lower, indicating strong generalization with minimal overfitting. Similarly, validation precision closely mirrored the steady improvement observed in training precision, suggesting the model’s effectiveness in minimizing false positives across both datasets. Regarding recall, the training recall showed a continuous upward trajectory, demonstrating the model’s capacity to capture true positives. Validation recall showed improvement overall. However, slight fluctuations occurred in later epochs. These may reflect variations in unseen data distribution or possible class imbalances. Regarding loss, training loss steadily decreased throughout the process, reflecting the model’s efficiency in learning from the data, whereas validation loss dropped sharply in the early epochs before stabilizing, signifying strong generalization during the initial stages and the model’s ability to avoid overfitting.

These comparisons highlight the consistency between training and validation trends across all metrics, emphasizing the DNN’s reliability in predicting unseen data. The model successfully balanced effective learning from the training data with robust performance on the validation set, underscoring its ability to achieve generalizable and reliable predictions. This balance between learning and generalization, evident across all four metrics, demonstrates the robustness of the DNN in achieving stable and accurate performance.

### 3.3 Comparison of model for outcome prediction

Five traditional machine learning models were employed to evaluate performance, each with distinct characteristics and applications. Logistic regression (LR) is a statistical method widely used for binary classification tasks, modeling the relationship between features and class probabilities through a sigmoid function (28). Decision trees (DT) construct a flowchart-like structure where decisions are made at nodes based on feature values, offering interpretability and flexibility in handling diverse data types (32). Naive Bayes (NB) is a probabilistic classifier based on Bayes’ theorem that assumes independence among features and is effective for tasks like text classification (33). Random forest (RF), an ensemble learning method, combines multiple decision trees to improve accuracy and robustness by aggregating their predictions (34). Support vector machines (SVM) identify an optimal hyperplane to separate classes, excelling in high-dimensional datasets and adaptable for nonlinear problems with kernel functions (35). These models provide a comprehensive baseline for performance evaluation in machine learning tasks. All the traditional models were implemented in Python (version 3.8.0). The scikit-learn library (version 0.24.2) of the Python machine learning package was used for RF, LR, DT, NB, and SVM. The TensorFlow library (version 2.5.0) was used for the DNN. The model training and prediction processes were completed with the Python (version 3.8.0), with all data processing steps handled via the pandas library (version 1.1.5) and NumPy library (version 1.19.5).

Tables 2, 3 show the evaluation results of each model. The DNN model consistently outperformed the other models across all datasets and metrics, meeting the criteria for “referable diabetic retinopathy prediction models” as defined in the Guidelines (section “Evaluation of ophthalmic artificial intelligence prediction models”) (31). The differences were significant for all datasets (*p* < 0.05) in the ROC curve comparisons. Regarding overall prediction, the DNN model performed 0.769 or better in the diabetic group with or without retinopathy (accuracy, 0.778; precision, 0.783; recall, 0.769; F1 score, 0.776; AUC, 0.833), outperforming most traditional machine learning models. This high level of accuracy suggests that the DNN is highly effective at managing complex features and nonlinear patterns, particularly when working with large datasets and high-dimensional inputs.

**TABLE 2:** Comparative performance of DNN, LR, DT, NB, RF, and SVM in predicting positive (“+”) and negative (“−“) classes.

Variable	Missing value(+)						Missing value(−)					
	DNN	LR	DT	NB	RF	SVM	DNN	LR	DT	NB	RF	SVM
Accuracy	0.752	0.741	0.652	0.876	0.754	0.344	0.773	0.766	0.619	0.367	0.757	0.656
Precision	0.769	0.761	0.632	0.582	0.757	0.752	0.756	0.746	0.639	0.747	0.754	0.631
Recall	0.752	0.741	0.652	0.876	0.754	0.517	0.773	0.766	0.619	0.367	0.757	0.829
F1 Score	0.760	0.751	0.642	0.699	0.756	0.612	0.764	0.756	0.629	0.493	0.756	0.716

DNN, deep neural network; LR, logistic regression; DT, decision trees; NB, naive bayes; RF, random forests; SVM, support vector machines.

**TABLE 3:** Comparative performance of DNN, LR, DT, NB, RF, and SVM for overall prediction.

Variable	DNN	LR	DT	NB	RF	SVM
Accuracy	0.778	0.753	0.630	0.718	0.758	0.776
Precision	0.783	0.761	0.571	0.710	0.767	0.776
Recall	0.769	0.741	0.636	0.738	0.743	0.776
F1 Score	0.776	0.751	0.602	0.724	0.755	0.776
AUC	0.833	0.822	0.631	0.769	0.829	0.831

AUC, area under the curve; DNN, deep neural network; LR, logistic regression; DT, decision tree; NB, naive Bayes; RF, random forest; SVM, support vector machine. *p* < 0.05 for both groups.

**FIGURE 4:**
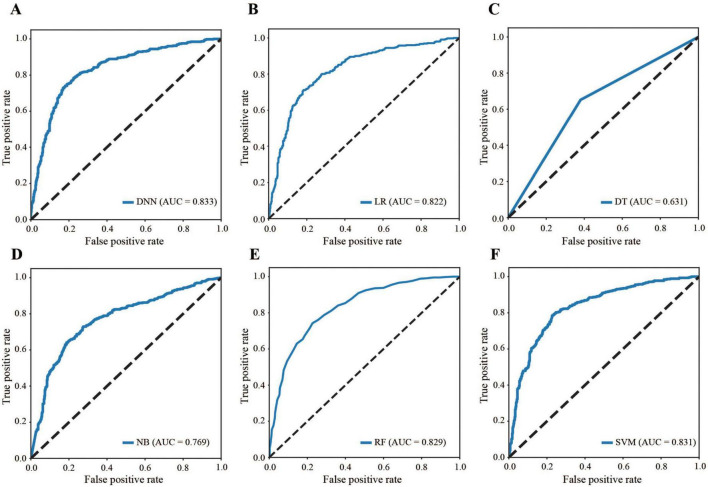
Comparison of ROC curves. **(A)** DNN, **(B)** LR, **(C)** DT, **(D)** NB, **(E)** RF, **(F)** SVM. ROC: receiver operating characteristic, DNN: deep neural network, LR: logistic regression, DT: decision tree, NB: naive Bayes, RF: random forest, SVM: support vector machine, AUC: area under the curve.

Figure 4 compares the ROC curves of all the models across the dataset, highlighting that the DNN model’s curve was consistently above the others (AUC: 0.833 for DNN, 0.822 for LR, 0.631 for DT, 0.769 for NB, 0.829 for RF, and 0.831 for SVM), demonstrating superior performance (Table 3). The AUC of 0.833 falls within the performance range as defined by the Guidelines (31). Conversely, models such as SVM and DT display flatter curves, underscoring their limitations in capturing complex nonlinear relationships.

### 3.4 Assessment and interpretation of the models

**FIGURE 5:**
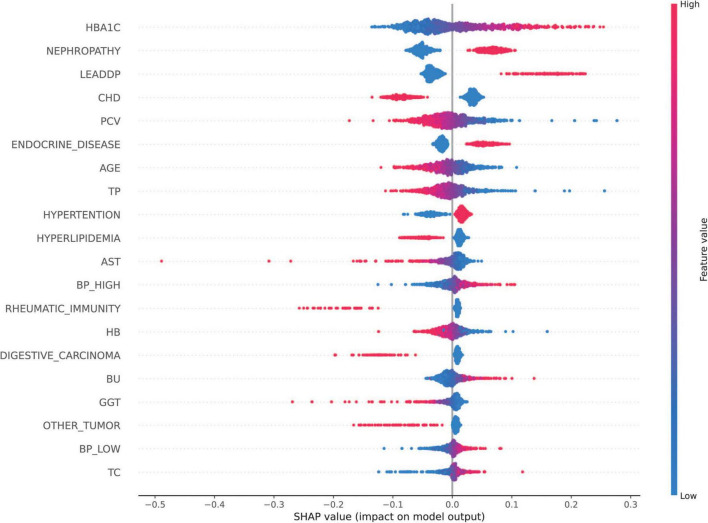
SHAP Feature Importance Map. SHAP values of each feature on the diabetes complications prediction model are shown, with features listed in descending order of importance. The horizontal coordinate is the SHAP value, which indicates the effect of the feature on the model output (positive values indicate a positive effect and negative values indicate a negative effect); the vertical coordinate is the name of the feature.

**FIGURE 6:**
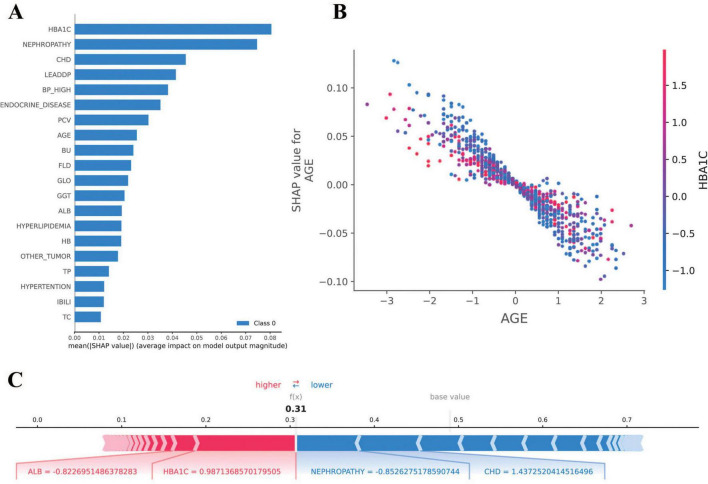
Results of SHAP analysis. **(A)** SHAP feature importance plot: demonstrates the mean SHAP value [mean(|SHAP value|)] of each feature for the diabetes complications prediction model. **(B)** SHAP dependency plot: demonstrates the relationship between AGE (age) and SHAP value, indicating that the effect of age on the risk of diabetic complications is nonlinearly distributed. **(C)** SHAP force diagram: demonstrates the interpretation of SHAP values for a sample of individuals. A base value of 0.5 indicates the default predictive probability of the model in the absence of feature information.

The SHAP analysis was employed to interpret the model’s predictions, aligning with the Guidelines’ emphasis on model interpretability evaluation (section “Evaluation of artificial intelligence model development in ophthalmology”) (31). As shown in Figure 5, the results revealed that HbA1c and nephropathy were the most significant predictors, with SHAP values validated against clinical reference standards for feature importance in diabetic retinopathy. Cardiovascular-related features, such as CHD, LEADDP, and BP_HIGH, also demonstrated substantial contributions, aligning with clinical evidence that links cardiovascular health to diabetic complications (36–38). Additionally, features like age, TP, and hyperlipidemia showed moderate influences, further enriching the model’s predictive capability. Figure 6 illustrates the SHAP dependence plots, which highlighted non-linear relationships, such as the impact of age on complication risk, while individual force plots provided transparent explanations for specific predictions. Overall, the SHAP analysis not only validated the model’s alignment with established clinical knowledge but also enhanced its interpretability, offering valuable insights into the key drivers of diabetic complications and supporting its potential for clinical decision-making.

## 4 Discussion

In this retrospective cohort study based on the Diabetes Complications dataset, we developed a deep learning model to evaluate the future risk of diabetes and diabetic retinopathy and compared its performance with that of traditional statistical models. To our knowledge, this is the first study using deep learning to predict both diabetes and its associated retinopathy complications in a real-world context. We observed that the deep learning model outperformed five conventional machine learning methods across all classification tasks according to five commonly used evaluation metrics.

As one of the most prevalent microvascular complications, DR affects visual function in 14.77% to 22.43% of diabetic individuals in China (39). The pressing need to offer targeted advice on preventing and managing DR underscores the importance of examining the factors contributing to its occurrence. Numerous studies have explored the risk factors for DR across various populations and clinical samples (40–43). As demonstrated by previous research, the complexity of DR arises from various factors influencing both diabetes and DR development, such as the duration of diabetes, blood glucose levels, HbA1c, and hypertension (43, 44). These factors align with our study’s findings, as significant differences were identified between diabetic patients with and without retinopathy regarding key variables such as HbA1c levels, blood pressure, and nephropathy rates (Table 1). The statistical analyses performed on the DCDS data using chi-square tests and independent samples *t*-tests provided robust evidence supporting these associations. Moreover, our DNN-based predictive model for DR leveraged these and other variables to effectively extract complex patterns and enhance prediction accuracy, demonstrating the relevance of these factors in both statistical and machine-learning contexts. This integrated approach underscores the importance of combining traditional statistical methods with advanced machine learning techniques to deepen our understanding of DR’s multifactorial nature. Studies such as those by Oh et al. showed that the LASSO model achieved an AUC of 81%, surpassing traditional metrics such as fasting glucose (AUC 54%) and glycosylated hemoglobin (AUC 69%) for diagnosing DR (45). Additionally, by comparing multiple machine learning algorithms, Tsao et al. identified insulin use and diabetes duration as key factors in determining high-risk patients for DR (42).

Given the numerous variables that contribute to DR occurrence, a substantial sample size is necessary to analyze risk factors thoroughly and build accurate predictive models. Deep neural networks are advantageous over traditional machine learning algorithms when dealing with large data sets and high-dimensional data. For example, logistic regression, a linear model, is prone to distortions in weight estimation when independent variables are highly correlated (46). On the other hand, the XGBoost algorithm, based on decision trees, is a nonparametric estimation method and does not suffer from the same issue, although its predictive performance is typically inferior to that of deep neural networks (47).

The rapid accumulation of extensive medical datasets has been fueled by the establishment of large-scale cohort studies involving tens of thousands to millions of participants worldwide. These datasets offer powerful opportunities to address complex health questions beyond the limitations of traditional clinical and observational research. Their vastness and ease of processing critical information, such as mortality rates and disease registries, enable the identification of previously unknown risk factors and statistically significant associations with disease incidence (48–50). For this study, such advantages guided the choice of the PHDA as the data source. Its comprehensive and high-quality datasets, designed to reflect real-world conditions, were particularly suited to exploring diabetes and its complications. This alignment between the PHDA’s focus and the study objectives ensured the robustness and applicability of the findings, providing a meaningful foundation for our analyses.

Additionally, the advent of machine learning has addressed the shortcomings of conventional risk prediction methods that rely on traditional regression analysis (51). Unsupervised deep learning models identify relevant patterns through weight and bias adjustments. This process occurs automatically, enabling detection of subtle patterns. Such patterns might escape notice in conventional human analysis. These models have the computational power to simultaneously evaluate a broad range of variables. Our DNN model utilized a straightforward architecture with two hidden layers, yet achieved clinically meaningful performance (AUC = 0.833) in predicting diabetic retinopathy risk. The model’s strength lies in its ability to integrate routinely collected clinical variables into an automated screening tool, facilitating early detection of high-risk patients through pattern recognition that surpasses conventional prediction methods. While this demonstrates the potential for improved risk stratification, future studies should explore more diverse deep-learning architectures to further optimize predictive accuracy and enable personalized treatment strategies.

Future efforts should aim to enhance model performance and interpretability by improving dataset diversity to ensure generalizability across different populations, incorporating interpretable elements into deep learning architectures to support clinical decision-making, and establishing mechanisms that allow the model to dynamically update as clinical practice patterns evolve. These strategies would help address current limitations while maintaining the model’s strong predictive capacity.

## 5 Study limitations

Our study’s findings should be interpreted considering several potential limitations. First, while our dataset specifically identified DR cases, it did not stratify by clinical severity stages (e.g., nonproliferative vs. proliferative DR) or distinguish referable/vision-threatening DR [as defined by international standards (52)]. This may affect the clinical interpretability of predictions, as the model treats all DR cases as a homogeneous group despite varying intervention needs.

We did not perform external validation to assess the reproducibility or generalizability of our results. While the individual cohorts were validated by dividing them into development and validation datasets, prediction accuracy may still decline when applying the model to cohorts from diverse regions, ethnicities, countries, or healthcare settings. Future studies should prioritize external validation using multicenter datasets to address this challenge, ensuring broader applicability and reliability across varying contexts.

Next, since the specific risk factors contributing to the events remain unidentified, concrete recommendations for managing these factors are currently infeasible. Future research should explore hybrid approaches that integrate interpretable models with deep learning techniques, allowing for a balance between prediction accuracy and the identification of actionable risk factors. These approaches could help tailor interventions and improve outcomes in real-world applications.

Third, while internal validation showed promising results, the model requires external validation using independent datasets from diverse healthcare settings to confirm generalizability.

Finally, as the training data were derived from a single national registry, the model’s performance may vary when applied to populations with different demographic characteristics or healthcare practice patterns. Additionally, the lack of granular DR staging data limits the model’s utility for triaging patients by clinical urgency. Future studies should validate these findings across multiple regions and healthcare systems while incorporating standardized DR severity classifications.

## 6 Conclusion

DR, a major microvascular complication of DM, presents considerable challenges for early detection and effective management, particularly due to the limitations of traditional screening methods. By leveraging advanced machine learning techniques on high-quality big data, we demonstrated that a DNN-based approach significantly outperforms five conventional statistical methods in predicting retinopathy among diabetic patients. Future studies should focus on developing predictive models with diverse deep-learning techniques. These models can accurately identify diabetic retinopathy cases. Such capability will enable personalized treatments and optimal resource allocation for high-risk patients.

## Data Availability

Publicly available datasets were analyzed in this study. This data can be found at: https://www.ncmi.cn//phda/dataDetails.do?id=CSTR:A0006.11.A0005.201905.000282 (National Center for Population Health Sciences Data Warehouse, Diabetes Complications Data Set).

## Footnotes

1
http://creativecommons.org/publicdomain/zero/1.0/

2
https://www.ncmi.cn//phda/dataDetails.do?id=CSTR:A0006.11.A0005.201905.000282
